# Evaluation of the Adsorption Performance and Sustainability of Exfoliated Graphite Nanoplatelets (xGnP) for VOCs

**DOI:** 10.3390/ma8115412

**Published:** 2015-11-11

**Authors:** Seong Jin Chang, Seunghwan Wi, Su-Gwang Jeong, Sumin Kim

**Affiliations:** School of Architecture, Soongsil University, Seoul 156-743, Korea; tjdwls329@ssu.ac.kr (S.J.C.); dnltmdghks@ssu.ac.kr (S.W.); wjdtnrhkd@ssu.ac.kr (S.-G.J.)

**Keywords:** volatile organic compounds (VOCs), exfoliated graphite nanoplatelets (xGnP), adsorption performance, thermal extractor analysis, indoor air quality

## Abstract

Exfoliated graphite nanoplatelets (xGnP), which combine the layered structure and low price of nanoclays with the superior mechanical, electrical, and thermal properties of carbon nanotubes, are very cost-effective, and can simultaneously provide a multitude of physical and chemical property enhancements. In this study, we evaluated xGnP’s adsorption performance of volatile organic compounds (VOCs) according to thermal extractor (TE) analysis for seven days in order to use the xGnP as an adsorption material of pollutants. In addition, we carried out a sustainability evaluation in order to evaluate its adsorption capacity over 28 days. The results indicate that the adsorption performance of xGnP is higher than for other adsorption materials such as zeolite. Also, we determined that the adsorption performance of xGnP is maintained continuously for 28 days and that its adsorption capacity is large.

## 1. Introduction

Among all pollutants, building materials play a major role in determining the indoor air quality due to their large surface area and permanent exposure to indoor air. Building materials can release a wide range of pollutants, particularly volatile organic compounds (VOCs), which can degrade indoor air quality, making it worse than that of outdoor air [1]. Recent studies of VOC emissions in four newly built, unoccupied test houses showed that the building materials are the main source of indoor air pollution [2]. Polymeric materials that emit a lot of VOCs are used widely in the construction, decorating, and furnishing of homes, offices, and schools, as well as other non-industrial work places. Some constitute large surface areas within buildings, such as coatings and coverings on walls, ceilings, and floors [3]. VOCs are often noxious or carcinogenic, either directly or indirectly, posing many severe environmental problems such as adverse effects on human health at very low concentrations [4]. Various studies have proposed some common methods for the removal of VOCs, including thermal oxidation, adsorption, condensation, membrane separation, and biological treatment [5,6,7,8,9,10]. Among these methods, adsorption has been proven to be an effective method for the removal of VOCs because it is fast, safe, and economically feasible [5].

Inorganic and natural materials can be used for the adsorption of VOCs. Zeolites and activated carbons, which are inorganic materials, are the most commonly used for the adsorption of VOCs and are an effective means of collecting these harmful compounds [11]. These adsorption materials of VOCs are substantially porous, and graphite is also a porous carbon material and its specific surface area and pore volume can be expanded by using the shape modification process [1]. Recently, studies examining exfoliated graphite nanoplatelets have been conducted, and Thostenson *et al.* carried out research for the ultrasonicated-ozone modification of exfoliated graphite (EG) [12]. Exfoliated graphite nanoplatelets (xGnP) are comprised of a graphitic carbon-based material. xGnP that are less than 10 nm thick and have a diameter of 15 μm are made through acid treatment, blasting, and shredding processes [13,14]. Most of the adsorption materials such as zeolite, diatomite, and active carbon have already been evaluated, but not xGnP. The measurement method for evaluating the adsorption of VOCs is done according to ISO/DIS 16000-23, ISO/DIS 16000-24, JIS A 1905-1, JIS A 1905-2, JIS A 1906 [15,16,17,18,19,20]. The adsorption ratio after seven days and the cumulative adsorption amount over seven days is measured. The methods also measure the re-emission for three days. The adsorption performance of the adsorbent material is evaluated by the foregoing measurement results. However, because of differences in the adsorption capacities of adsorption materials, a long-term performance evaluation is necessary.

In this study, we evaluated the adsorption performance of xGnP and other adsorption materials using thermal extractor analysis for seven days. In addition, we conducted a sustainability evaluation in order to evaluate the adsorption capacity of the adsorption materials for 28 days.

## 2. Experimental Section

### 2.1. Materials

**Figure 1:**
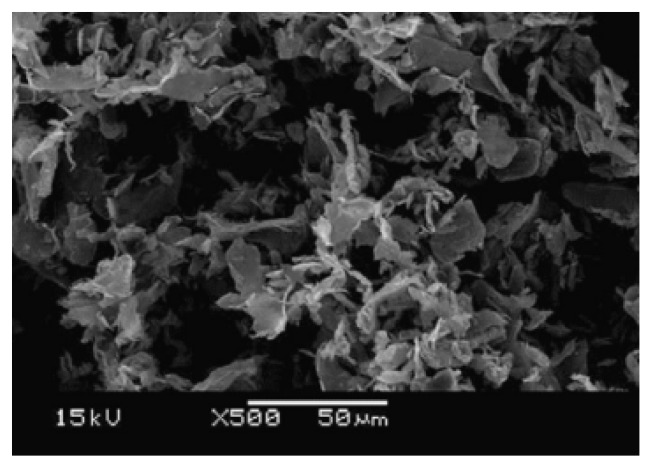
Scanning electron microscop (SEM) images of Exfoliated graphite nanoplatelets (xGnP).

xGnP was prepared from sulphuric acid-intercalated expandable graphite (3772) (obtained from Asbury Graphite Mills, NJ, USA), by applying a cost- and time-effective exfoliation process that was initially proposed by Drzal’s group. xGnP, which combines the layered structure and low price of nanoclays with the superior mechanical, electrical, and thermal properties of carbon nanotubes, is very cost-effective, and can simultaneously provide a multitude of physical and chemical property enhancements [21,22]. Zeolite and perlite are porous materials and offer excellent adsorption performance of the pollutants. In this study, they are used for comparison with xGnP, and both natural and improved zeolites (JST-MS100, Daejin Chemical, Namyang-Ju, Korea) were used. Table 1 sets out the specific surface area (m^2^·g^−1^) and total pore volume (cm^3^·g^−1^) of the four samples by Brunauer Emmett Teller (BET) analysis, and Figure 1 shows the Scanning electron microscop (SEM) images of xGnP.

**Table 1:** The result of Brunauer Emmett Teller (BET) analysis.

Samples	Specific Surface Area (m^2^·g^−1^)	Total Pore Volume (cm^3^·g^−1^)
xGnP	20.4	0.082
Perlite	3.3	0.004
Zeolite (Natural)	23.4	0.031
Zeolite (JST-MS100)	755.6	0.352

### 2.2. Methods

#### 2.2.1. Thermal Extractor (TE)

The seven-day evaluation and the sustainability evaluation were conducted through thermal extractor (TE) analysis. TE analysis is used mainly to measure the total VOC (TVOC) and formaldehyde emitted from construction materials such as medium density fiberboard (MDF), particle board (PB), paints, and adhesives. TE analysis can be used with very small samples to measure pollutant emissions, and the test can be run according to temperature because the samples can raise the temperature by 190 °C. The test samples were conditioned before testing at 50% ± 5% relative humidity (RH) for at least 24 h and sealed in aluminum foil [1]. 

**Figure 2:**
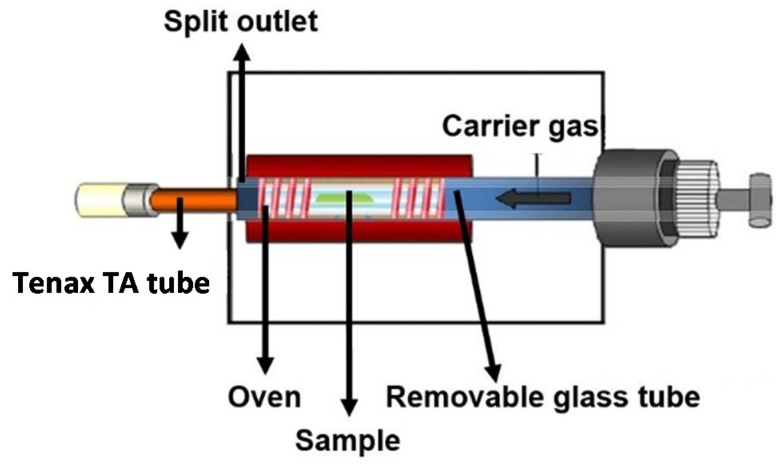
Schematic diagram of the thermal extractor (TE).

Figure 2 shows a schematic diagram of the thermal extractor (TE). The TE consists of an adjustable oven heating a glass tube with a sample inside. Adsorbed samples were placed in a glass extraction tube. The adsorbed sample size was limited, both by the diameter of the tube and by the heatable length of the oven, to a maximum of 70 mm. The VOCs were purged under a pure nitrogen gas stream at a constant flow on a Tenax TA tube and 2,4-DNPH (dinitrophenylhydrazine) cartridge; 2,4-DNPH, followed by high performance liquid chromatography (HPLC) analysis, is a widely used selective and sensitive method for the measurement of carbonyl compounds in air. In normal use, the entire gas flow passes over the adsorbent material. The sampling volume was 1 L [1,23]. The VOC concentrations were analyzed by using GC-MSD. GC-MSD is a gas chromatography (GC, Agilent-6890N, National Instrumentation Center for Environmental Management, Seoul, Korea)-mass spectrum detector (MSD, Agilent-5975, National Instrumentation Center for Environmental Management).

#### 2.2.2. Evaluation of Adsorption Performance

We conducted a comparative experiment with xGnP, zeolite, and perlite. The comparative experiment is a simple experiment, other than the method specified in ISO/DIS 16000-23, ISO/DIS 16000-24, JIS A 1905-1, JIS A 1905-2, JIS A 1906, used in order to evaluate only the adsorption performance of xGnP. First, 10 g samples of xGnP, zeolite (both natural and JST-MS100), and perlite were prepared. The prepared samples were placed in a constant temperature and humidity chamber that was maintained at 25 °C and 50% humidity, respectively. Then, a source emitting VOCs was introduced to the chamber at 24-h intervals. After seven days, the pollutants adsorbed on each sample were analyzed by thermal extractor (TE) analysis. Additionally, a sustainability evaluation was conducted for 28 days under the same conditions.

## 3. Results and Discussion

### 3.1. Adsorption Performances of the Samples over Seven Days

This experiment can be compared to the adsorption performance in the same experimental conditions. Table 2 shows the adsorption performances of the samples over seven days. After seven days, the TVOC adsorption amount of perlite was 150.48 μg/m^3^, which was the highest amount among the four samples. Next, the TVOC adsorption amount of xGnP was 128.93 μg/m^3^. The lowest TVOC adsorption amount was that of natural zeolite, at 9.99 μg/m^3^. This value indicates that the TVOC adsorption ability of xGnP is 12.91 times larger than that of natural zeolite. The 5VOC (benzene, toluene, ethyl benzene, xylene, styrene) adsorption amount of xGnP was 25.08 μg/m^3^. Although the TVOC adsorption amount of xGnP was not the highest, its 5VOC adsorption amount was the highest. Although the experimental conditions were not based on international standards, this result indicates that the adsorption performance of xGnP is higher than that of existing adsorption materials.

**Table 2:** Result of adsorption performance for seven days.

Samples	Zeolite (JST-MS100)	Zeolite (Natural)	Perlite	xGnP
TVOC (μg/m^3^)	76.91	9.99	150.48	128.93
Benzene (μg/m^3^)	2.17	1.71	2.69	4.37
Toluene (μg/m^3^)	2.07	1.61	10.38	18.83
Ethyl Benzene (μg/m^3^)	-	-	-	-
Xylene (μg/m^3^)	-	-	-	1.89
Styrene (μg/m^3^)	-	-	-	-
5VOC (μg/m^3^)	4.25	3.33	13.07	25.08
5VOC (%)	5.52	33.13	8.69	19.45

### 3.2. Sustainability Evaluation for 28 Days

**Figure 3:**
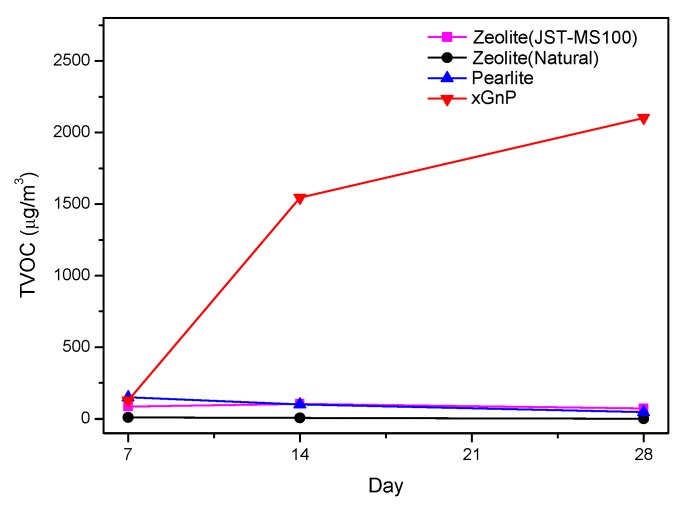
TVOC adsorption amount of xGnP for 28 days.

**Figure 4:**
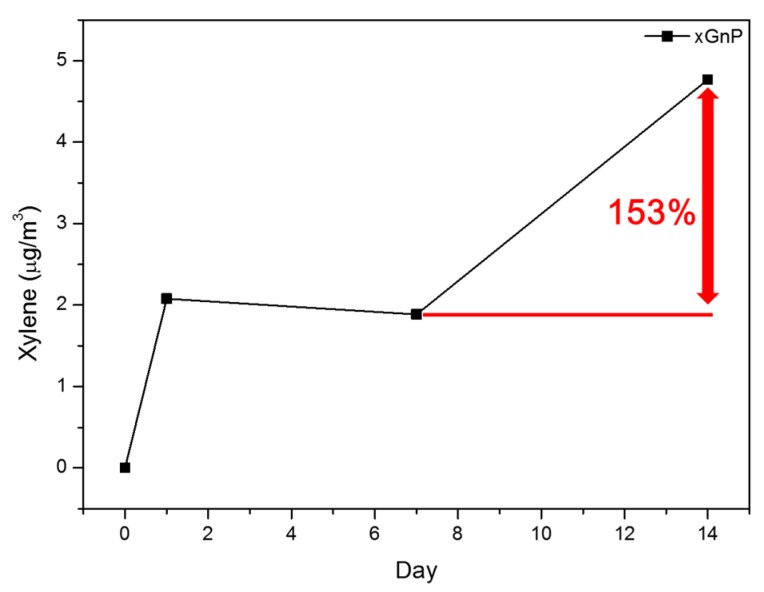
Xylene adsorption amount of xGnP.

Figure 3 and Table 3 show the sustainability of the samples’ adsorption performance. As previously mentioned, the TVOC adsorption amount of perlite was the highest after seven days, with xGnP the next highest. However, during the second week, the TVOC adsorption amount of xGnP increased from 128.93 to 1545.31 μg/m^3^ on the 14th day. This was the highest adsorption amount among the four samples on the 14th day. In addition, xGnP’s adsorption of xylene increased by 153% during the second week. Figure 4 shows the xylene adsorption performance of xGnP. On the other hand, perlite, which showed the highest performance at seven days, was reduced from 150.48 to 101.95 μg/m^3^ on the 14th day. Over the entire sustainability evaluation period of 28 days, the TVOC adsorption amount of zeolite (JST-MS100) increased from 86.96 μg/m^3^ at seven days to 105.50 μg/m^3^ on the 14th day, and then fell back to 72.49 μg/m^3^ on the 28th day. Natural zeolite and perlite were decreased continuously from seven to 28 days. The TVOC adsorption amount of xGnP only tended to increase steadily. This result shows that xGnP is more advantageous than existing adsorption materials over this longer period of time.

**Table 3:** Sustainability evaluation of adsorption performance.

TVOC	7 days (μg/m^3^)	14 days (μg/m^3^)	28 days (μg/m^3^)
Zeolite (JST-MS100)	86.96	105.50	72.49
Zeolite (Natural)	9.99	7.09	2.27
Perlite	150.48	101.95	48.20
xGnP	128.93	1545.31	2102.64

## 4. Conclusions

In this study, we evaluated the adsorption performance of xGnP for seven days. In addition, we carried out a sustainability evaluation in order to evaluate its adsorption capacity for 28 days.

On the basis of the obtained results, we concluded that the 5VOC adsorption amount of the xGnP showed the highest performance. Although our experimental conditions were not based on international standards, this result indicates that the adsorption performance of xGnP is higher than those of existing adsorption materials such as zeolite. Furthermore, the result of the sustainability evaluation was that the TVOC adsorption amount of xGnP only tended to increase steadily for 28 days. In other words, we determined that the adsorption performance of xGnP is maintained continuously and that its adsorption capacity is large. This result indicates that it is possible to discover promising new materials which are not necessarily highlighted during a seven-day evaluation. Therefore, it is necessary to consider new experimental methods for sustainability evaluations of adsorption performance.

We will later evaluate the TVOC adsorption performance of xGnP according to ISO/DIS 16000-23, ISO/DIS 16000-24, JIS A 1905-1, JIS A 1905-2, JIS A 1906, and we will study new experimental methods for sustainability evaluations of adsorption performance.

## References

[1] Lee J.H., Kim S. (2012). The determination of the adsorption performance of graphite for VOCs and formaldehyde. Energy Build..

[2] Yu C., Crump D. (1998). A review of the emission of VOCs from polymeric materials used in buildings. Build. Environ..

[3] Choi D.H., Kang D.H., Kim S.S., Yeo M.S., Kim K.W. (2010). The impact of a non-adhesive floating installation method on emissions and indoor concentrations of VOCs. Indoor Built. Environ..

[4] Qu F., Zhu L., Yang K. (2009). Adsorbtion behaviors of volatile organic compounds (VOCs) on porous clay heterostructures (PCH). J. Hazard. Mater..

[5] Hsu L.J., Lin C.C. (2012). Binary VOCs absorption in a rotating packed bed with blade packings. J. Environ. Manag..

[6] Lalanne F., Malhautier L., Roux J.C., Fanlo J.L. (2008). Absorption of a mixture of volatile organic compounds (VOCs) in aqueous solutions of soluble cutting oil. Bioresour. Technol..

[7] Heymes F., Demoustier P.M., Charbit F., Fanlo J.L., Moulin P. (2006). A new efficient absorption liquid to treat exhaust air loaded with toluene. Chem. Eng. J..

[8] Dwivedi P., Gaur V., Sharma A., Verma N. (2004). Comparative study of removal of volatile organic compounds by cryogenic condensation and adsorption by activated carbon fiber. Sep. Purif. Technol..

[9] Ji W., Sikdar S.K., Hwang S.T. (1994). Modeling of multicomponent pervaporation for removal of volatile organic compounds from water. J. Membr. Sci..

[10] Daubert I., Lafforgue C., Maranges C., Fonade C. (2001). Feasibility study of a compact process for biological treatment of highly soluble VOCs polluted gaseous effluent. Biotechnol. Prog..

[11] Kim K.J., Ahn H.G. (2012). The effect of pore structure of zeolite on the adsorption of VOCs and their desorption properties by microwave heating. Microporous Mesoporous Mater..

[12] Rider A.N., An Q., Thostenson E.T., Brack N. (2014). Ultrasonicated-ozone modification of exfoliated graphite for stable aqueous graphitic nanoplatelet dispersions. Nanotechnology.

[13] Kim S., Do I., Drzal L.T. (2009). Multifunctional exfoliated graphite nanoplatelets-LLDPE nanocomposites fabricated by solution compounding method and various screw rotating systems. Macromol. Mater. Eng..

[14] Kalaitzidou K., Fukushima H., Drzal L.T. (2007). Mechanical properties and morphological characterization of exfoliated graphite-polypropylene nanocomposites. Compos. Part A Appl. Sci. Manuf..

[15] Kim J.H., Kim H.J., Yoon D.W. (2011). Evaluation studies on the adsorption VOCs performance of the natural porous materials for the development of absorptive building materials. J. KIAEBS.

[16] International Organization for Standardization (2008). Indoor Air—Part 23: Performance Test for Evaluating the Reduction of Formaldehyde Concentrations by Sorptive Building Materials.

[17] International Organization for Standardization (2008). Indoor Air—Part 24: Performance Test for Evaluating the Reduction of Volatile Organic Compounds and Carbonyl Compounds without Formaldehyde Concentrations by Sorptive Building Materials.

[18] Japan Industrial Standard (2007). Performance Test of Sorptive Building Materials of Reducing Indoor Air Pollution with Small Chamber—Part 1: Measurement of Adsorption Flux with Supplying Constant Concentration of Formaldehyde.

[19] Japan Industrial Standard (2007). Performance Test for Sorptive Building Materials of Reducing Indoor Air Pollution with Small Chamber—Part 2: Measurment of Capability for Suppressing Formaldehyde Emission.

[20] Japan Industrial Standard (2008). Performance Test of Sorptive Building Materials of Reducing Indoor Air Pollution with Small Chamber—Measurement of Adsorption Flux with Supplying Constant Concentration of Contaminant Air of VOC and Aldehydes without Formaldehyde.

[21] Wolkoff P. (2011). Photocopiers and indoor air pollution. Atmos. Environ..

[22] Kim S., Drzal L.T. (2009). High latent storage and high thermal conductive phase change materials using exfoliated graphite nanoplatelets. Sol. Energy Mater. Sol. Cells.

[23] Lee Y.K., Kim H.J. (2012). The effect of temperature on VOCs and carbonyl compounds emission from wooden flooring by thermal extractor test method. Build. Environ..

